# Evaluating the Modified Barthel Index for Policy and Practice in Reablement: Lessons From Australia's Short‐Term Restorative Care Program

**DOI:** 10.1111/ajag.70136

**Published:** 2026-02-04

**Authors:** Luke Schmidt, Daniel Broszczak, Margaret MacAndrew, Christina Parker

**Affiliations:** ^1^ BallyCara Ltd Scarborough Queensland Australia; ^2^ School of Biomedical Sciences, Centre for Biomedical Technologies, Faculty of Health Queensland University of Technology Kelvin Grove Queensland Australia; ^3^ Tissue Repair and Translational Physiology Group, School of Biomedical Sciences, Faculty of Health Queensland University of Technology Kelvin Grove Queensland Australia; ^4^ School of Nursing, Centre for Healthcare Transformation, Faculty of Health Queensland University of Technology Kelvin Grove Queensland Australia

**Keywords:** activities of daily living, aged, disability evaluation

## Abstract

**Objective:**

The Modified Barthel Index (MBI) is the sole reporting metric required by the Australian Government for the Short‐Term Restorative Care program (STRC). This study investigated the suitability of the MBI as an outcome measure of functioning/self‐care ability in the older Australian reablement context.

**Methods:**

This was a retrospective cohort study where historical data from 921 participants involved in the STRC between January 2018 to March 2023 were collected from an aged care provider located in Australia. This study compared STRC program responders and non‐responders based on MBI across a range of demographic variables. Additionally, Generalised Linear Modelling was performed to investigate the utility of the MBI to inform changes to the delivery of the intervention.

**Results:**

Although the MBI was able to show significant differences between responders and non‐responders at baseline (*p* ≤ 0.05), this is likely due to the sample size used. It was identified that the MBI suffers from a ceiling effect in the studied population. Therefore, the ability of the MBI to inform evidence‐based changes relating to program delivery is limited.

**Conclusions:**

This study provides a solid evidence base to guide the implementation of assessments in future programs and studies. This is due to the identification of limitations of the sole reporting metric used in the program. Based on findings throughout this manuscript, a range of standardised assessments dependent on the participant's goals should be implemented in future programs, such as the Restorative Care Pathway in the Support at Home program.

## Policy Impact

This study highlights the importance of implementing standardised, sensitive and specific assessments in restorative care programs. With the Restorative Care Pathway (RCP) to replace the Short‐Term Restorative Care Program (STRC) in late 2025, findings from the STRC, like those presented in this paper, should inform the implementation of the RCP.

## 1 Introduction

Reablement and restorative care programs for older adults are short‐term, goal‐oriented interventions designed to help individuals regain or maintain the skills and confidence for independent living [1, 2]. These programs typically focus on improving physical function such as ambulation, self‐care and other activities of daily living depending on the needs of the participant [2]. Most often, these programs are implemented following an acute event, such as illness, injury or hospitalisation, which are known to have a negative effect on physical functioning [3]. Through improving physical functioning, these programs aim to reduce long‐term reliance on healthcare services, therefore reducing the overall burden on primary health care [1]. Reablement differs from traditional care approaches in that it emphasises promoting autonomy rather than providing ongoing assistance [2]. As new evidence emerges, these programs must continually adapt and evolve to ensure the programs are effective in responding to the changing needs of older adults [4]. However, if these alterations are based on data gathered from sub‐optimal measures for the population and/or effect of the reablement program, the needs of older adults will not be met.

In Australia, a reablement/restorative care program that is currently being reviewed for modification is the Short‐Term Restorative Care program (STRC). The STRC is an Australian Government‐funded, eight‐week multidisciplinary intervention that aims to reduce or slow functional decline in older adults (aged 65 years or older). Largely, the implementation of the program is based on the needs of the consumer in a client‐centred approach, with any accredited allied health profession able to provide services to meet the goals of the consumer. The allied health professions provide intervention in a coordinated manner that aligns with the consumer's goals. The STRC is transitioning to the Restorative Care Pathway (RCP) in the new Support at Home program [5, 6]. The Australian Government has indicated the eligibility criteria, aims and model of the RCP will largely remain the same, with the delivery of the intervention, including the composition of allied health team and number of sessions per allied health profession, being modified by the coordinating provider based on the consumer's needs and goals [5, 6]. Throughout the lifespan of the STRC, which was announced in the 2015–2016 Australian Federal Budget and will run until November 2025 [5, 6], the only measure of success collected by the Australian Government is the total score of the Modified Barthel Index (MBI) from pre‐ and post‐intervention as a measure of baseline and exit functioning/self‐care ability [6]. Aged care providers that coordinate and deliver the STRC have also conducted their own assessments of participant progress including those related to physical and cognitive/psychosocial outcomes to ensure a more comprehensive assessment of the intervention [7, 8, 9].

Generally, interventions such as the STRC, which are restorative or reablement‐focussed, multidisciplinary and intensive in nature, tend to involve an exercise component [10, 11]. Such programs can lead to improvements in motivation for the allied health team and empower the consumer [12, 13], while also facilitating improved physical functioning [14, 15]. However, similar programs have generally been found to be ineffective for reducing mortality [16, 17], emergency department visits [16] or hospitalisations [16, 17], and ineffective in improving quality of life [14, 16, 17]. Reablement programs have also been reported to be difficult to implement in a holistic and sufficiently client‐centred manner [2]. To date, although the literature regarding the STRC is limited, it has been identified that it can lead to significant improvements in physical and psychological outcomes [7, 8, 9]. Interestingly, although all STRC literature to this point has reported that MBI scores increase following STRC intervention [7, 8, 9], the suitability of the MBI as the sole reporting metric in the STRC has not been thoroughly investigated. Brett et al. [7] did, however, point out that the MBI can result in a ceiling effect and therefore may not be appropriate for use in the STRC. In the documentation for the RCP, although assessments are discussed, no detail has been provided about what assessments will be compulsory, if any [5, 18].

Based on the presented gap in the literature and the potential significance such a study may have to inform new programs, the overarching aim of this study was to expand on the observation by Brett et al. [7] and what is currently known about the only compulsory measure for the STRC, the MBI. Specifically, the aims of this study were to investigate the demographic and intervention delivery differences between responders (participants who demonstrated an improved MBI score) and non‐responders (participants who did not have an improved MBI score) of the STRC intervention to examine the utility of the MBI in assessing the STRC's ability to improve functioning. Such investigation would therefore inform the implementation of assessments in other similar programs to the STRC, such as the RCP. This manuscript highlights findings that may inform program documentation, and subsequently the implementation of assessments in restorative and reablement programs for older populations.

## 2 Methods

### 2.1 Design, Participants and Setting

The data presented in this paper were from a retrospective cohort study that has previously been described [9]. This study was conducted to investigate whether the STRC program led to changes in overall functioning and function/self‐care subdomains as determined by the MBI in older Australians who undertook the STRC program with an aged care provider located in Brisbane, Australia. This investigation was conducted via secondary analysis of clinical records using the largest dataset, which has been published on to date [9].

Participants needed both an entry and exit MBI assessment to be included in this study. Where this was not the case, the data for that participant were removed before analysis. The STRC program was delivered as set out by the Australian Government and included an initial and final assessment separated by eight weeks of individualised intervention delivered with a coordinated, multidisciplinary approach by qualified allied health professionals and engaging on a case‐specific basis. Full details of the program can be accessed via the program manual (https://www.health.gov.au/resources/publications/short‐term‐restorative‐care‐programme‐manual?language=en) and the current authors' previous publication [6, 9].

Following ethics approval, historical data were obtained from BallyCara Ltd. in a de‐identified format. BallyCara Ltd. (previously the Hibernian QLD Friendly Society) is a charitable organisation operating across Southeast Queensland and Melbourne, Victoria. BallyCara Ltd. has strategic ambitions to improve learning and research outcomes that will benefit their consumers.

### 2.2 Data Collection and Measures

All demographic, health and pre‐intervention data were collected in the first week of the program, and the post‐intervention data were collected in the final week (week eight) of the program. Data that were collected for use in this study included:

- demographic characteristics (sex, age, and general health conditions);
- program details (treating allied health team); and
- assessment outcomes (Modified Barthel Index [MBI] with associated functioning/self‐care ability subdomain data)

The MBI is a validated, 11‐item questionnaire that assesses the ability of an individual to care for themselves and complete activities of daily living [19]. Of the 11 items, only 10 items are used in a given assessment, with item three replacing Item 2 if the participant was unable to walk [19]. The 11 items assess subdomains of function, which are as follows: chair and bed transfers, ambulation, ambulation with a wheelchair (reported if unable to walk), stair climbing, toilet transfers, bowel control, bladder control, bathing, dressing, personal hygiene and grooming, and feeding [19]. Each of the subdomains is rated on a scale from 0–5, 0–10 or 0–15 depending on the subdomain, with the use of descriptors that detail the five levels of the ordinal scale [19]. The MBI's highest total score is 100, which indicates the highest level of function and independence [19]. The Australian Government only requires the MBI to be completed at pre‐ and post‐program delivery, with the total score subsequently reported to the Government [6].

De‐identified data were extracted from clinical records and collated in an Excel spreadsheet (.xlsx). Data were collected from all participants of the STRC program from the commencement of this program at BallyCara Ltd. (January 2018) to March 2023.

### 2.3 Statistical Analysis and Visualisation

All analysis were performed using SPSS v30.0 (IBM Corp., USA). Initially, the data were formatted and cleaned, with ineligible participants and values that were outside the range of the measure (i.e., data entry errors) removed from the dataset. Descriptive statistics were performed followed by assessment of normality. The Kolmogorov–Smirnov test was used; all continuous variables were not normally distributed, so nonparametric tests were utilised herein. Pre‐ to post‐intervention differences in functioning/self‐care ability using MBI total and subdomain scores were assessed by way of Wilcoxon Signed Rank tests. Bivariate differences in demographic data with relation to the participants' response to the intervention, with groups defined as responders (participants who demonstrated an increased MBI score of one or more) and non‐responders (participants who did not have an improved MBI score, either having no change or decreasing), were obtained by χ2 test (with Yates' Continuity Correction applied) and Mann–Whitney U analysis as appropriate. Given the homogeneity between the responders' and non‐responders' median and IQR values for many of the variables, it is salient to note that Mann–Whitney U analysis assesses the difference in rank sums between groups, not the median and IQR values, to ascertain statistical significance. Multivariate analysis of allied health team composition in relation to improvements in functioning was obtained by way of Generalised Linear Modelling (logit link function and binomial distribution; GLM). The allied health team composition was entered into the analysis as a binary variable, with each allied health profession either being in the treating team [yes] or not [no]. Statistical significance was accepted at *p* < 0.05 for all statistical tests except Mann–Whitney U and Wilcoxon Signed Rank tests, where following Bonferroni correction for multiple comparisons, statistical significance was accepted at *p* < 0.004. Violin plots were generated using Power BI v2.149.1429.0 (Microsoft, USA).

## 3 Results

### 3.1 Analysis of Participant Demographics and Baseline Data When Grouped by MBI Outcome

**FIGURE 1:**
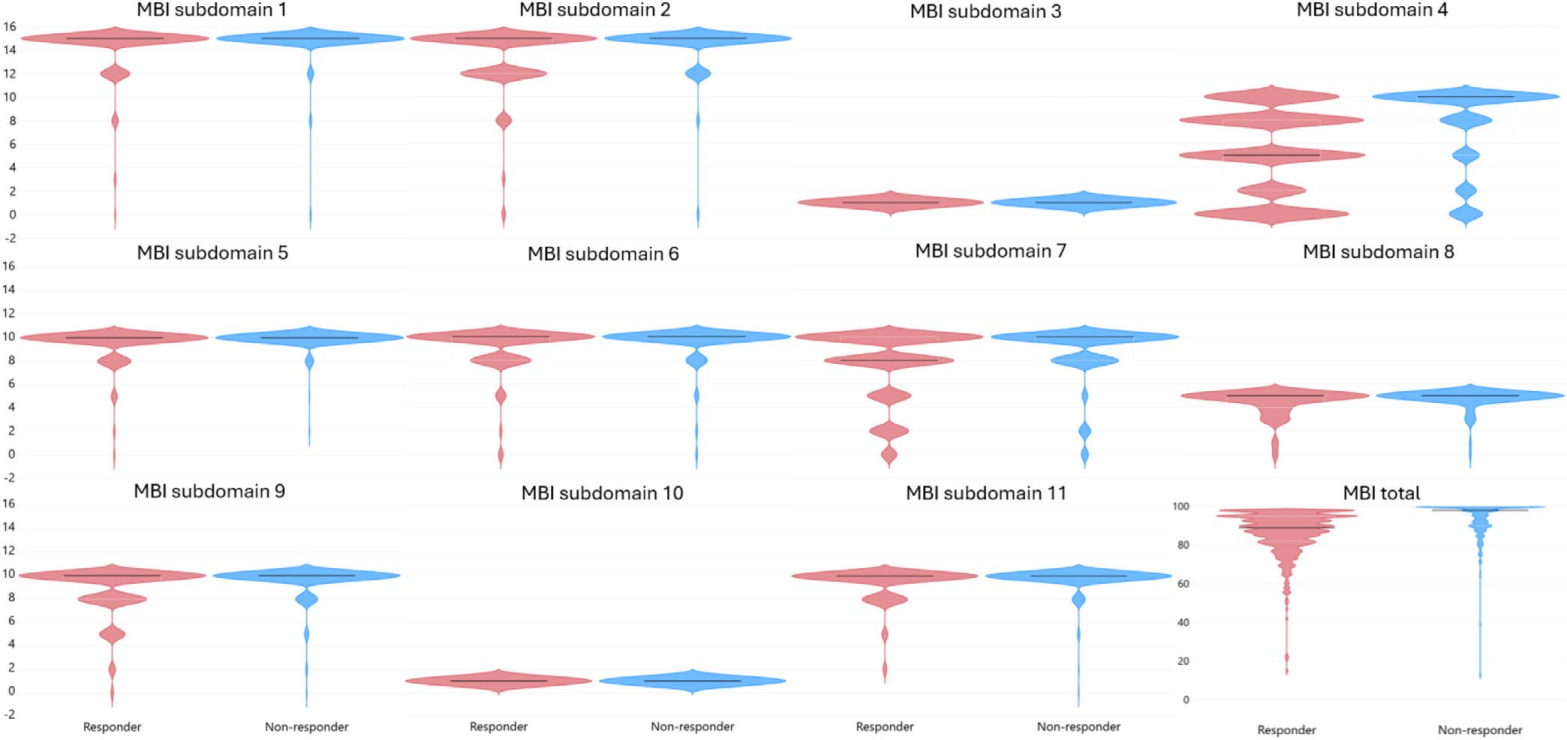
Violin plots demonstrate the differences between responders and non‐responders as assessed by the MBI subdomain and total scores at baseline that could not be observed with median and IRQ (Table 1) alone. Red = Responders; Blue = Non‐responders; Black line = median value; x axis = normalised number of responses for each rating separated by responder/non‐responder, y axis = rating response for each subdomain/total MBI score.

A total of 921 participants met the inclusion criteria for this study. At baseline, there were no significant differences between participants that improved (responders) or had no improvement or decreased total MBI outcome (non‐responders) post‐intervention for the variables of age, sex or diagnosis of medical condition (Table 1). There were, however, significant differences (*p* ≤ 0.001) in total MBI outcome based on all pre‐intervention MBI subdomain scores (except subdomain 10 following Bonferroni correction) and the pre‐intervention total MBI score. Importantly, and observed in Figure 1, the majority of the median scores demonstrate a ceiling effect in this population, and therefore, the significant result would only be possible for a large sample size. Analysis comparing pre‐ and post‐intervention MBI subdomain and total MBI scores demonstrates the same issues with sensitivity; as such, the result from this analysis is provided as Data S1. These results indicate that the MBI may not be appropriate for use in the STRC as it lacks sensitivity due to the ceiling effect observed in the STRC population.

**TABLE 1:** Demographic and baseline data comparing responders vs. non‐responders reported as number (%) or median ± IQR.

	Total (*n* = 921)	Responders (*n* = 565)	Non‐responders (*n* = 356)	*p* Value
Age (years)	79 ± 11	79 ± 11	78 ± 11	0.11
Sex (male)	361 (39.2)	217 (38.4)	144 (40.4)	0.58
**Co‐morbidities**				
Musculoskeletal condition	547 (59.4)	343 (74.7)	204 (73.9)	0.86
Cardiac condition	471 (51.1)	291 (63.4)	180 (65.2)	0.68
Neurological condition	198 (21.5)	128 (27.9)	70 (25.4)	0.51
Renal condition	22 (2.4)	10 (2.2)	12 (4.3)	0.15
Metabolic condition	169 (18.3)	108 (23.5)	61 (22.1)	0.72
Cancer	95 (10.3)	53 (11.5)	42 (15.2)	0.19
Respiratory condition	165 (17.9)	113 (24.6)	52 (18.8)	0.08
Mental health condition	106 (11.5)	69 (15)	37 (13.4)	0.62
**MBI at pre‐intervention**				
MBI subdomain 1 pre‐intervention (15‐point scale)	15 ± 0	15 ± 0	15 ± 0	≤ 0.001
MBI subdomain 2 pre‐intervention (15‐point scale)	15 ± 3	15 ± 3	15 ± 0	≤ 0.001
MBI subdomain 3 pre‐intervention (5‐point scale)	5 ± 5	5 ± 5	5 ± 0	≤ 0.001
MBI subdomain 4 pre‐intervention (10‐point scale)	8 ± 8	5 ± 6	10 ± 5	≤ 0.001
MBI subdomain 5 pre‐intervention (10‐point scale)	10 ± 0	10 ± 0	10 ± 0	≤ 0.001
MBI subdomain 6 pre‐intervention (10‐point scale)	10 ± 0	10 ± 2	10 ± 0	≤ 0.001
MBI subdomain 7 pre‐intervention (10‐point scale)	10 ± 2	8 ± 2	10 ± 2	≤ 0.001
MBI subdomain 8 pre‐intervention (5‐point scale)	5 ± 0	5 ± 1	5 ± 0	≤ 0.001
MBI subdomain 9 pre‐intervention (10‐point scale)	10 ± 2	10 ± 2	10 ± 0	≤ 0.001
MBI subdomain 10 pre‐intervention (5‐point scale)	5 ± 0	5 ± 0	5 ± 0	0.006
MBI subdomain 11 pre‐intervention (10‐point scale)	10 ± 0	10 ± 0	10 ± 0	≤ 0.001
MBI total pre‐intervention (out of 100)	92 ± 13	89 ± 13	98 ± 10	≤ 0.001

*Note:* MBI subdomain 1 = Chair/bed transfers; MBI subdomain 2 = Ambulation; MBI subdomain 3 = Ambulation/wheelchair (reported if unable to walk); MBI subdomain 4 = Stair climbing; MBI subdomain 5 = Toilet transfers; MBI subdomain 6 = Bowel control; MBI subdomain 7 = Bladder control; MBI subdomain 8 = Bathing; MBI subdomain 9 = Dressing; MBI subdomain 10 = Personal hygiene/Grooming; MBI subdomain 11 = Feeding.

Abbreviation: MBI, Modified Barthel Index.

### 3.2 Modelling Allied Health Team Composition Impact on Total MBI Score

Using a GLM, at the main effects level of analysis (Table 2), the inclusion of exercise physiology in the team was the only allied health profession that resulted in a significant differentiation between responders and non‐responders in regard to total MBI outcome (*p* ≤ 0.001). When considering the design of the intervention, additive synergies between allied health professions may result in greater improvements in total MBI outcome; as such, the interaction effects analysis of the GLM should also be considered (Table 3). The interaction effects analysis returned 14 allied health teams that resulted in statistically significant improvements in total MBI outcome, all of which included exercise physiology, although no team resulted in a greater Wald χ2 value than exercise physiology alone in the main effects analysis. This indicates that when using the MBI to group participants as responders and non‐responders, the primary reason for undertaking a multidisciplinary intervention (the synergies between the allied health professions leading to greater outcomes) was unable to be appropriately assessed. Teams, rather than an individual allied health profession, should have resulted in greater outcomes for participants. This finding adds further evidence that the MBI is not an appropriate measure for use in the STRC.

**TABLE 2:** GLM main effects of single allied health professions on total MBI outcome, controlling for age.

Health professions (number of times included in allied heath team)	β	Standard error	Lower 95% Wald confidence interval	Upper 95% Wald confidence interval	Wald χ2	*p* Value
Occupational therapy (*n* = 905)	0.616	0.5711	−0.503	1.735	7.915	0.28
Physiotherapy (*n* = 852)	0.046	0.2795	−0.501	0.594	0.028	0.87
Exercise physiology (*n* = 884)	1.356	0.3938	0.584	2.128	11.854	≤ 0.001
Speech therapy (*n* = 64)	3.77	0.2881	−0.188	0.942	1.714	0.19
Dietician (*n* = 202)	0.221	0.1763	−0.124	0.567	1.573	0.21
Podiatry (*n* = 177)	−0.039	0.1808	−0.394	0.315	0.048	0.83
Nursing (*n* = 610)	−0.200	0.1520	−0.498	0.098	1.732	0.19
Social work (*n* = 431)	0.56	0.1433	−0.225	0.337	0.155	0.69
Counselling/wellness coaching (*n* = 12)	1.030	0.7835	−0.506	2.565	1.727	0.19
Massage therapy (*n* = 12)	−0.398	0.6014	−1.577	0.780	0.439	0.51

**TABLE 3:** GLM interaction effects between allied health professions on total MBI outcome, controlling for age.

β	Standard error	Lower 95% Wald confidence interval	Upper 95% Wald confidence interval	Wald χ2	*p* Value	Occupational therapy	Physiotherapy	Exercise physiology	Speech therapy	Dietician	Podiatry	Nursing	Social work	Counselling/wellness coaching	Massage therapy
0.048	0.0198	0.009	0.087	5.906	0.015	✓	✓	✓	✓	✓	X	✓	✓	X	X
0.033	0.0158	0.002	0.064	4.440	0.04	✓	✓	✓	✓	✓	X	✓	X	X	X
0.024	0.0121	0.001	0.048	4.040	0.04	✓	✓	✓	X	✓	X	✓	✓	X	X
0.032	0.0115	0.009	0.054	7.481	0.006	✓	✓	✓	X	✓	X	✓	X	X	X
0.030	0.0133	0.004	0.056	5.078	0.02	✓	✓	✓	X	✓	X	X	✓	X	X
0.035	0.0134	0.009	0.061	6.936	0.008	✓	✓	✓	X	✓	X	X	X	X	X
0.030	0.0114	0.008	0.053	7.032	0.008	✓	✓	✓	X	X	✓	✓	✓	X	X
0.027	0.0116	0.004	0.050	5.370	0.02	✓	✓	✓	X	X	✓	✓	X	X	X
0.026	0.0132	0.000	0.052	3.904	0.048	✓	✓	✓	X	X	✓	X	✓	X	X
0.021	0.0106	0.000	0.042	3.883	0.049	✓	✓	✓	X	X	X	✓	✓	X	X
0.021	0.0103	0.001	0.041	4.069	0.04	✓	✓	✓	X	X	X	✓	X	X	X
0.027	0.0110	0.005	0.048	5.801	0.02	✓	✓	✓	X	X	X	X	✓	X	X
0.025	0.0106	0.004	0.046	5.596	0.02	✓	✓	✓	X	X	X	X	X	X	X
0.042	0.0148	0.013	0.071	7.940	0.005	✓	X	✓	X	X	X	✓	✓	X	X

*Note:* ‘✓’ = part of treating team in that particular model; ‘X' with shading = not part of treating team in that particular model.

## 4 Discussion

This study investigated the suitability of the MBI as an outcome measure of functioning/self‐care ability in an older Australian reablement context. Such investigation supports evidenced‐based decisions and will assist in informing the development and implementation of other similar programs to the STRC, like the RCP. The current manuscript highlights findings that may inform program documentation, implementation, and delivery of similar interventions.

Conducting appropriate assessments to investigate which population may benefit most from initiatives, such as the STRC, is important for ensuring future programs optimise participant outcomes. In the current study, it was found that age, sex and type of medical condition the participants had were not statistically associated with being a responder or non‐responder to the STRC intervention as assessed by the MBI. The presented result, achieved with dichotomous data, is underscored by Brett et al. [7], who reported no association between sex and age with MBI when analysed as a continuous variable. A related finding has also previously been reported by the current authors who found that there were no statistically significant differences for baseline MBI between males and females [9]. It was, however, identified in the current study that lower total and subdomain MBI scores, and therefore lower functioning/self‐care ability, were significantly associated with being a responder to the STRC intervention (Table 1). From these results, one could presume that an intervention designed to improve functioning/self‐care ability would be more beneficial to those with a lower degree of functioning/self‐care ability. An alternative view based on closer observation of the data is that, although there are statistical differences, there is little variation between responders and non‐responders to the intervention (except for Subdomain 4 and 7) as demonstrated by identical median values and identical or near‐identical IQR values (Table 1, Figure 1). These median values are the maximum score possible for each of the subdomains (excluding responders for Subdomain 4 and 7). This indicates that the MBI is not appropriate for use in the STRC, or similar interventions, due to a ceiling effect being observed in the majority of eligible participants at the subdomain level. Based on this finding, and how the scores are summed to achieve a total MBI score, it would be logical to presume that the total MBI score also suffers from a lack of sensitivity.

Disability and functional decline are multifaceted, and thus, an appropriate measure to assess a multifaceted intervention should be able to shed light on a number of facets relating to intervention delivery. The STRC is a multifaceted intervention as it is multidisciplinary, allowing multiple allied health professions to work with the participant across the eight‐week intervention to help them meet their goals and slow or reverse functional decline and disability progression. To analyse the synergies that should exist between allied health professions in the STRC, GLM analysis was used. When investigating the main effects on MBI outcome, controlling for age, exercise physiology was the only allied health profession that was identified as having a statistically significant effect on whether the participant was a responder or non‐responder to the STRC intervention (Table 2). On review of the interactions, again controlling for age, it was found that the multidisciplinary team composition that resulted in the greatest number of responders included an Exercise Physiologist, an Occupational Therapist, a Nurse and a Social Worker (Table 3). Model comparisons showed that including exercise physiology in the intervention, without accounting for synergy with other allied health professions, significantly increased the number of responders. This appears at odds with the multidisciplinary approach central to the STRC. However, as noted, the MBI appears to lack sensitivity in this context. This highlights the need for more tailored and comprehensive assessments aligned with participants' goals to more accurately evaluate the STRC's multidisciplinary impact. Certainly, the provider from whom the data were obtained conducts their own assessments beyond the MBI to be able to ensure the efficacy of their STRC intervention. However, as this is not mandated in the delivery of the STRC, it is beyond the scope of this study.

## 5 Limitations

The study design allowed for a large and highly representative population, which facilitates generalisability of results in the Australian context. However, due to the secondary analysis of clinical records, it is acknowledged that care must be taken in the interpretation of the results. Care was taken to avoid overinterpretation of the presented results. Due to limits in computing power, the GLM analysis was conducted as binary. Such an analysis, although able to discriminate between responders and non‐responders, is unable to discern magnitude of response which is acknowledged as an important aspect of a participant's response to an intervention.

## 6 Conclusions

In conclusion, based on the data presented, this study provides a solid evidence base to guide the implementation of assessments in future programs and studies. This is due to the identification of the limitations of the MBI as the sole metric used in the STRC. Based on findings outlined throughout this manuscript, consideration should be given to implementing a range of standardised assessments dependent on the participant's goals. These assessments should be well‐established measures of physical and psychological functioning that have the sensitivity and specificity to determine whether there are changes in areas of function that are important to the quality of life of the individual participant. Without such a change to future iterations of the STRC (i.e., the RCP in the Support at Home program), evidence‐based amendments to the intervention based on suboptimal measures could be misleading.

Given the findings in this manuscript, future directions include the investigation of assessments used by providers nationwide in the provision of the STRC. Furthermore, a cost–benefit analysis of the STRC using an appropriate outcome measure would allow for comparisons with future programs such as the RCP.

## Ethics Statement

Human Research Ethics Committee (HREC) approval was obtained from Queensland University of Technology HREC (ethics approval number: 7338) and complies with the guidelines set out in the Declaration of Helsinki. This ethics approval waived the requirement of individual consent for use of the historical data set in a de‐identified format.

## Conflicts of Interest

L.S. was employed by BallyCara Ltd. as an Exercise Physiologist and Research Officer during intial data collection and manusucript drafting. No other conflicts of interest declared.

## Supporting information


**Data S1:** ajag70136‐sup‐0001‐Supinfo.docx.

## Data Availability

Research data are not shared as per ethics application.
