# Supplementary material for: Evaluating the Modified Barthel Index for Policy and Practice in Reablement: Lessons From Australia's Short‐Term Restorative Care Program
Source: Australas J Ageing. 2026 Feb 4;45(1):e70136. doi: 10.1111/ajag.70136 (PMC12872200; doi:10.1111/ajag.70136)
Supplement: Supplementary file 1 — Data S1: ajag70136‐sup‐0001‐Supinfo.docx. [file AJAG-45-0-s001.docx]

**Impact of STRC on functioning and self-care ability as assessed by MBI**

All subdomains of the MBI, except for subdomain 3 (Ambulation/wheelchair - reported if participant was unable to walk), reported a statistically significant improvement post-intervention by way of Mann-Whitney U analysis (*p* ≤ 0.001; **Supplemental Table 1**). Furthermore, there was a statistically significant increase in the total MBI score (*p* ≤ 0.001). However, as stated previously the majority of median scores for all measures demonstrate a ceiling effect in this population. This leads to a large overlap in the IQR beyond the total MBI median scores from pre- to post- intervention. These results indicate that the MBI does not have utility in the STRC for assessing the overall efficacy of the program.

**Supplemental Table 1:** Change in functioning and self-care ability as assessed by comparing pre- and post-intervention scores on the MBI at subdomain and total score levels (median ± IQR)

|  | **Pre-intervention** | **Post-intervention** | ***p* Value** |
| --- | --- | --- | --- |
| MBI subdomain 1 | 15 ± 0 | 15 ± 0 | ≤0.001 |
| MBI subdomain 2 | 15 ± 3 | 15 ± 0 | ≤0.001 |
| MBI subdomain 3 | 5 ± 5 | 5 ± 0 | >0.99 |
| MBI subdomain 4 | 8 ± 8 | 8 ± 5 | ≤0.001 |
| MBI subdomain 5 | 10 ± 0 | 10 ± 0 | ≤0.001 |
| MBI subdomain 6 | 10 ± 0 | 10 ± 0 | ≤0.001 |
| MBI subdomain 7 | 10 ± 2 | 10 ± 2 | ≤0.001 |
| MBI subdomain 8 | 5 ± 0 | 5 ± 0 | ≤0.001 |
| MBI subdomain 9 | 10 ± 2 | 10 ± 0 | ≤0.001 |
| MBI subdomain 10 | 5 ± 0 | 5 ± 0 | ≤0.001 |
| MBI subdomain 11 | 10 ± 0 | 10 ± 0 | ≤0.001 |
| MBI total | 92 ± 13 | 96 ±10 | ≤0.001 |

Abbreviations: MBI, Modified Barthel Index.

MBI subdomain 1 = Chair/bed transfers; MBI subdomain 2 = Ambulation; MBI subdomain 3 = Ambulation/wheelchair (reported if unable to walk); MBI subdomain 4 = Stair climbing; MBI subdomain 5 = Toilet transfers; MBI subdomain 6 = Bowel control; MBI subdomain 7 = Bladder control; MBI subdomain 8 = Bathing; MBI subdomain 9 = Dressing; MBI subdomain 10 = Personal hygiene/Grooming; MBI subdomain 11 = Feeding

**Supplemental discussion**

Further consideration of the total MBI score descriptive statistics at pre- and post-intervention (**Supplemental Table 1**) revealed a lack of sensitivity in the subdomains, impacting the overall sensitivity of the total score, as indicated by a median score within four units of each other and IQR values that overlap the median scores. These results reflect those observed by Brett et al (2025), who reported median total MBI scores of 91 and 95 at pre- and post-STRC intervention respectively.*^1^* These results are in contrast to that obtained by Falland et al (2024) who reported median MBI scores of 78.5 and 95 at pre- and post-STRC intervention, respectively.*^2^* These differences may be due to sample size differences, as the study by Falland et al (2024) reported a sample size of n = 62, and the current study, and the study by Brett et al (2025), have sample sizes of n > 480.*^1, 2^* This inconsistency in sensitivity is also reported in literature relating specifically to the MBI. The MBI was reported to increase the sensitivity of the Barthel Index (BI) in a stroke population.*^3^* Others indicated that this modified scale was no more sensitive than the original 20-point scale in the same clinical population.*^4^* Although, more recently, and again in a stroke population, the MBI has been verified as being more sensitive than the BI.*^5^* To this point, the MBI may be more sensitive than the BI, however, these comparisons have been conducted only in stroke populations, therefore the MBI may not be reliable, valid or sensitive in other clinical populations, explaining the differences in the STRC literature and limiting its utility in the STRC. Based on these findings, it is the position of the current authors, that the MBI should not be the sole reporting metric of the STRC or other similar interventions. Consideration should be given to implementing a range of standardised assessments dependant on the participant’s goals, that are well established measures of physical and psychological functioning that have the sensitivity and specificity to determine if there are changes in these areas.

1. Brett L, Collins A, Lemsing K, Poulos CJ. Client characteristics and outcomes of the Australian short-term restorative care programme: a cohort study. BMC Health Services Research. 2025;25(1):1-15.

2. Falland L, Henwood T, Keogh JW, Davison K. Prioritising restorative care programs in light of current age care reform. Australasian Journal on Ageing. 2024;43(1):191-8.

3. Shah S, Vanclay F, Cooper B. Improving the sensitivity of the Barthel Index for stroke rehabilitation. Journal of clinical epidemiology. 1989;42(8):703-9.

4. Hocking C, Williams M, Broad J, Baskett J. Sensitivity of Shah, Vanclay and Cooper's modified Barthel index. Clinical rehabilitation. 1999;13(2):141-7.

5. Wang Y-C, Chang P-F, Chen Y-M, Lee Y-C, Huang S-L, Chen M-H, et al. Comparison of responsiveness of the Barthel Index and modified Barthel Index in patients with stroke. Disability and Rehabilitation. 2023;45(6):1097-102.
